# Association of *MTHFR* C677T and A1298C polymorphisms with non-Hodgkin lymphoma susceptibility: Evidence from a meta-analysis

**DOI:** 10.1038/srep06159

**Published:** 2014-08-22

**Authors:** Jing He, Xiao-Yu Liao, Jin-Hong Zhu, Wen-Qiong Xue, Guo-Ping Shen, Shao-Yi Huang, Wei Chen, Wei-Hua Jia

**Affiliations:** 1 State Key Laboratory of Oncology in South China, Department of Experimental Research, Collaborative Innovation Center for Cancer Medicine, Sun Yat-Sen University Cancer Center, Guangzhou, Guangdong 510060, China; 2 Molecular Epidemiology Laboratory and Laboratory Medicine, Harbin Medical University Cancer Hospital, Harbin, Heilongjiang 150040, China; 3 Department of Radiation Oncology, The First Affiliated Hospital, Sun Yat-Sen University, Guangzhou, Guangdong 510080, China; 4 Department of Neurosurgery, The Second Affiliated Hospital, Sun Yat-Sen University, Guangzhou, Guangdong 510120, China

## Abstract

Methylenetetrahydrofolate reductase (MTHFR) is an important enzyme involved in folate metabolism and DNA synthesis. A number of studies have examined the association of *MTHFR* C677T and A1298C polymorphisms with non-Hodgkin lymphoma (NHL) susceptibility; however, the conclusions were contradictory. We searched available publications assessing the polymorphisms of *MTHFR* and NHL susceptibility from MEDLINE, EMBASE and CBM. Genotype-based mRNA expression analysis was performed using data from 270 individuals with three different ethnicities. Ultimately, a total of 7448 cases and 11146 controls from 25 studies were included for the C677T polymorphism, 6173 cases and 9725 controls from 19 studies for the A1298C polymorphism. Pooled results indicated that neither C677T nor A1298C polymorphism was associated with NHL susceptibility. However, C677T polymorphism showed a statistically significantly increased risk for Caucasians, but a decreased risk for Asians in the subgroup analysis by ethnicity. The same variants may confer increased susceptibility to develop follicular lymphoma (FL). Moreover, A1298C polymorphism was associated with increased NHL risk for Asians. This meta-analysis indicated that C677T polymorphism was associated with altered NHL susceptibility for Caucasians, Asians and FL. Increased NHL risk was also shown for A1298C among Asians. These findings warrant validation in large and well-designed prospective studies.

Cancer has been recognized as one of the most formidable public health challenges, with estimates of nearly 12.7 million cancer cases and 7.6 million cancer deaths having occurred in 2008. Non-Hodgkin lymphoma (NHL) is the eighth most frequently diagnosed tumor type among men and the tenth among women worldwide^1^. Noteworthily, according to GLOBOCAN 2008 estimates, 355900 new cases and 191400 deaths might have occurred in 2008. There are a variety of different subtypes of NHL. Generally NHL is categorized into two major groups: B cell lymphomas and T cell lymphomas, with B cell lymphomas making up majority of NHL cases (about 85%). Diffuse large B-cell lymphoma (DLBCL) and follicular lymphoma (FL) are the two major subtypes of B cell lymphomas^2^,^3^. North America, Australia/New Zealand, and Northern, Western, and Southern Europe with the highest incidence rates, and South-Central and Eastern Asia and the Caribbean with the lowest incidence rates^1^. Certain immunodefective conditions (e.g., immunosuppression, Epstein-Barr virus and human immunodeficiency virus infections) as well as occupational exposures to herbicides and chlorinated organic compounds are the main risk factors for NHL^1^,^4^. Moreover, deficiency of nutrients (e.g., folate) related to one-carbon metabolism is also a well-established risk factors for NHL^5^, which has been reported to likely lead to immune responses impaired^6^.

Folate is an important coenzyme in DNA synthesis. Methylenetetrahydrofolate reductase (MTHFR) is one of the most critical enzymes involved in folate metabolism and DNA synthesis, which catalyses the conversion of 5, 10-methylenetetrahydrofolate to 5-methyltetrahydrofolate irreversibly^7^. Reduced MTHFR activity may play an inhibitory role on the 5-methyltetrahydrofolate pathway, and may lead to the accumulation of 5-methylenetetrahydrofolate, and consequentially methylation of dUMP to dTMP is decreased. Uracil can misincorporate into DNA when the methylation of dUMP to dTMP is deficient, consequentially results in DNA double-strand breaks and other anomalies, if not repaired which may lead to carcinogenesis^8^,^9^,^10^.

The *MTHFR* gene is located at chromosome 1p36.3. Among all the identified single nucleotide polymorphisms (SNPs) in this gene, C677T (Ala222Val, rs1801133) and A1298C (Glu429Ala, rs1801131), likely associated with reduced enzyme activity, have been widely investigated in a variety of diseases, such as psychiatric disorders^11^, congenital anomalies^12^, colorectal cancer^13^, and so on. Numerous studies have focused on the relationship between these two polymorphisms and NHL risk^14^,^15^,^16^,^17^,^18^,^19^,^20^,^21^,^22^,^23^,^24^,^25^,^26^,^27^,^28^,^29^,^30^,^31^,^32^,^33^,^34^,^35^,^36^,^37^,^38^,^39^, but the conclusions remain controversial. The discrepancies among studies may be ascribed to the relatively small sample size in each investigation as well as ethnicity difference. Therefore, we performed this meta-analysis using genotype data from all eligible investigations to provide a more precise evaluation of the association of *MTHFR* C677T and A1298C polymorphisms with NHL susceptibility.

## Results

### Study characteristics

**Figure 1:**
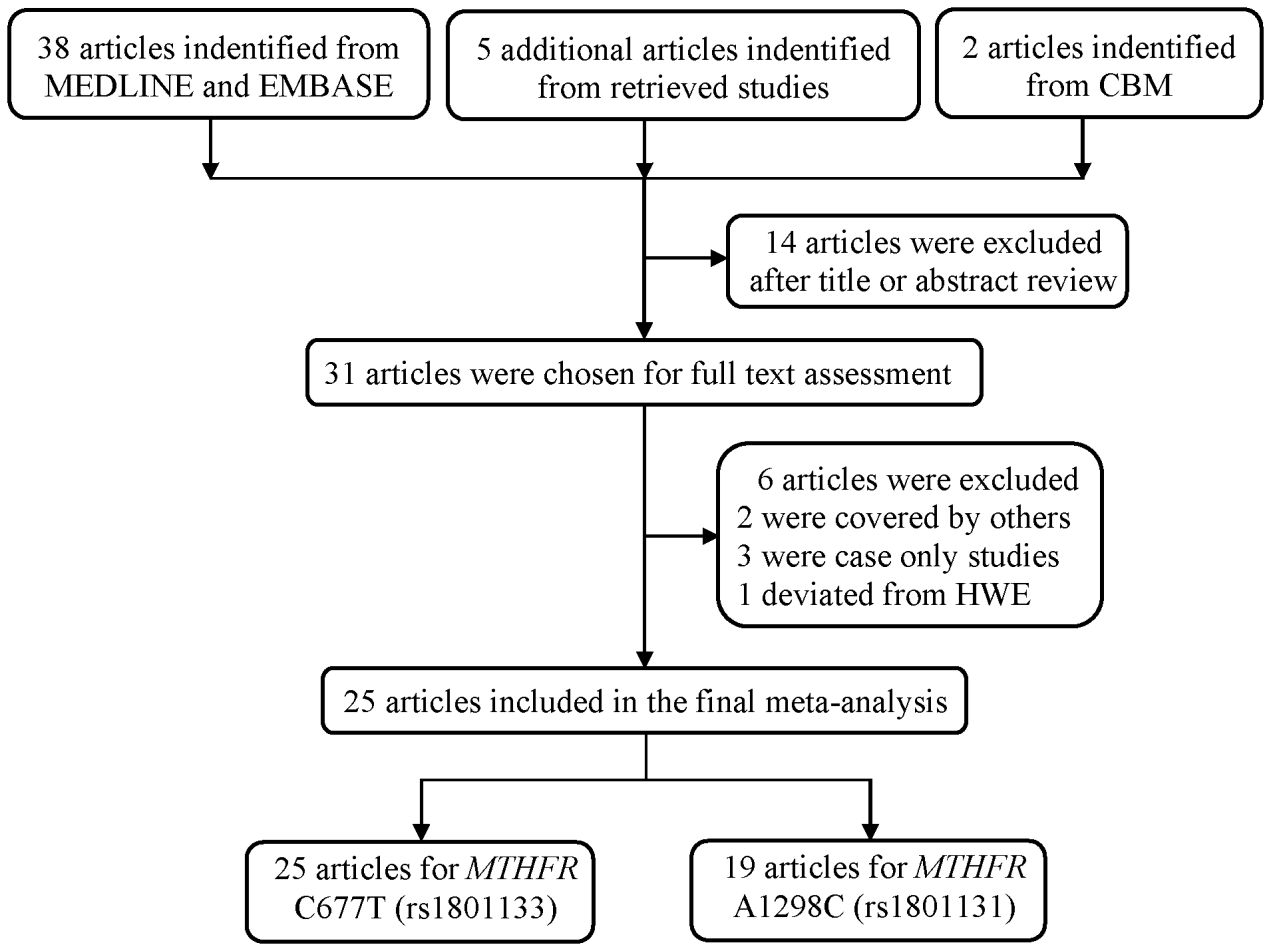
Flow diagram of selection of studies included in the current meta-analysis for the association between *MTHFR* gene polymorphisms and NHL susceptibility.

A total of 38 articles were initially indentified from MEDLINE and EMBASE, five more articles were indentified from the reference of retrieved studies, and ultimately, additional two articles were indentified from the CBM database (Figure 1). Of them, 14 articles were excluded after title and abstract assessment, while 31 articles met the crude inclusion criteria and were further evaluated. Among these remaining 31 publications, we further excluded two studies^15^,^26^ that were covered by other included investigations^20^,^24^, three case-only designed studies^40^,^41^,^42^, and one more study in which genotype frequency data in the controls for both of the C677T and A1298C polymorphisms were deviated from HWE (*P* = 0.009 for C677T and *P* = 0.040 for A1298C)^38^. Overall, 25 articles were included in the final meta-analysis. There were 25 articles with 7448 cases and 11146 controls for the C677T polymorphism, and 19 studies with 6173 cases and 9725 controls for the A1298C polymorphism (Table 1). Intriguingly, in the remaining studies, 12 and nine studies pertaining to C677T polymorphism provided detailed genotype frequency data for the DLBCL and FL subtype, respectively, while nine and eight studies regarding A1298C polymorphism provided detailed genotype data for the these two subtypes, respectively (Supplemental Table 1). Sample sizes of case in the incorporated studies ranged from 28 to 1103 for C677T polymorphism and from 31 to 1124 for A1298C polymorphism.

For the C677T polymorphism, there were 19 studies conducted in Caucasians, three studies in Asians, and three studies in mixed ethnic group. Of these studies, 18 were population based (PB) and seven were hospital based (HB) designed, respectively. Furthermore, 11 studies were considered as low quality (quality socre ≤ 9), and 14 (56%) were considered as high quality (quality score > 9). As to the A1298C polymorphism, there were 14 studies conducted in Caucasians, two studies in Asians, three studies in mixed ethnic group. While divided by the source of control, 15 studies were PB and four were HB. Among them, 6 were classified into low quality and 13 were classified into high quality. The mainly adopted genotyping methods were PCR-restriction fragment length polymorphism (12 and eight studies for C677T and A1298C, respectively) and Taqman (10 studies and eight studies for the C677T and A1298C, respectively).

### Meta-analysis results

**Figure 2:**
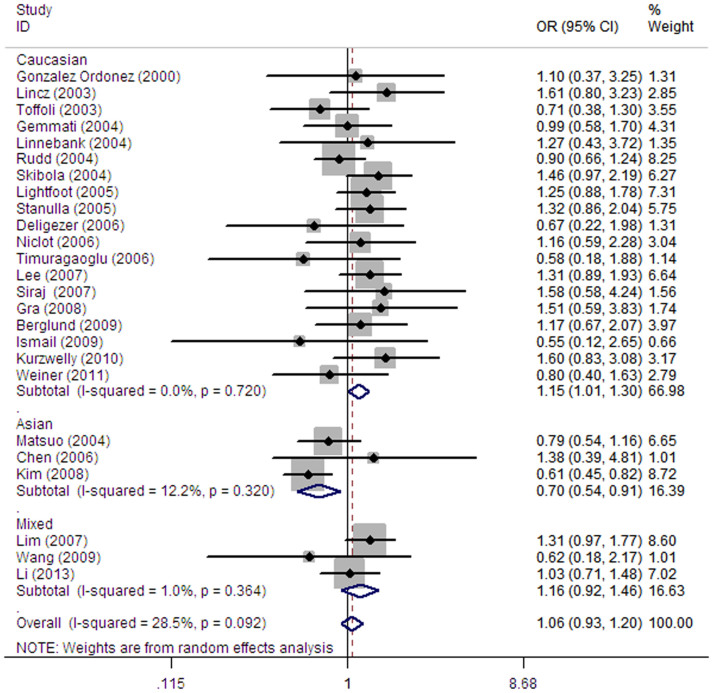
Forest plots of effect estimates for *MTHFR* C677T polymorphism and NHL susceptibility (TT vs. CC). For each study, the estimation of OR and its 95% CI are plotted with a box and a horizontal line. ◊, pooled ORs and its 95% CIs.

As shown in Table 2 and Figure 2, pooled analysis did not yield a significant association between *MTHFR* C677T polymorphism and overall NHL risk (homozygous: OR = 1.06, 95% CI = 0.93–1.20; heterozygous: OR = 0.97, 95% CI = 0.89–1.07; recessive: OR = 1.04, 95% CI = 0.95–1.15; dominant: OR = 0.99, 95% CI = 0.90–1.08 and allele comparing: OR = 1.01, 95% CI = 0.94–1.08). Next, we performed stratification analysis for the association between the C677T polymorphism variant genotypes by ethnicity, source of control, quality of studies, and tumor subtype. Stratification analysis by ethnicity revealed a statistically significantly increased NHL risk for Caucasians (homozygous: OR = 1.15, 95% CI = 1.01–1.30, and allele comparing: OR = 1.07, 95% CI = 1.01–1.13). In contrast, a significantly decreased risk of NHL was observed with the homozygous (OR = 0.70, 95% CI = 0.54–0.91) and dominant model (OR = 0.74, 95% CI = 0.59–0.91) genotypes for Asian group. Allele comparison further indicated that T variant allele is protective factor for NHL (T vs. C: OR = 0.81, 95% CI = 0.72–0.90). No significant association was found in the subgroup analysis by source of control and quality of studies. Moreover, stratification by tumor subtype demonstrated a significant increased risk of FL (homozygous: OR = 1.25, 95% CI = 1.01–1.53 and recessive: OR = 1.26, 95% CI = 1.04–1.53).

Similar to *MTHFR* C677T polymorphism, the presence of *MTHFR* A1298C polymorphism did not associate with an altered overall NHL risk (homozygous: OR = 1.21, 95% CI = 0.97–1.50; heterozygous: OR = 1.02, 95% CI = 0.95–1.09; recessive: OR = 1.21, 95% CI = 0.98–1.49; dominant: OR = 1.05, 95% CI = 0.96–1.14 and allele comparing: OR = 1.08, 95% CI = 0.98–1.18). Nevertheless, we observed a significantly increased risk of NHL for Asians (homozygous: OR = 1.54, 95% CI = 1.05–2.24; dominant: OR = 1.21, 95% CI = 1.03–1.42 and allele comparing: OR = 1.20, 95% CI = 1.05–1.38) but not other ethnic groups, when the analysis was stratified by ethnicity. No significant association was found in the remaining subgroup analyses by source of control, quality of studies and tumor subtype.

### The correlation between the mRNA expression and genotypes

The correlation between *MTHFR* mRNA expressions levels by the genotypes were explored for three ethnic groups (i.e., CEU, YRI and Asian) and the whole group (Table 3). No significant alteration in the mRNA expression levels was found for the C677T polymorphism under all the genetic models.

Interestingly, we found that C variant allele of *MTHFR* A1298C polymorphism significantly correlated with increased *MTHFR* mRNA expression levels among Caucasians (dominant model: *P* = 0.046), but decreased mRNA expression among Asians (heterozygous: *P* = 0.024 and dominant: *P* = 0.028).

### Heterogeneity and sensitivity analyses

As shown in Table 2, substantial heterogeneities were observed among all investigations for the C677T polymorphism and NHL risk (homozygous: *P* = 0.092; heterozygous: *P* = 0.027; dominant model: *P* = 0.006 and allele comparing: *P* = 0.006), except for the recessive model (*P* = 0.465). We also observed considerable heterogeneities for the A1298C polymorphism (homozygous: *P* < 0.001; recessive model *P* < 0.001; dominant model: *P* = 0.064 and allele comparing: *P* < 0.001), except for the heterozygous model (*P* = 0.471). The meta-regression analysis did not yield any significant difference between subgroup analysis. Thus, leave-one-out sensitivity analyses indicated that no single study could alter the pooled ORs obviously (data not shown).

### Publication bias

**Figure 3:**
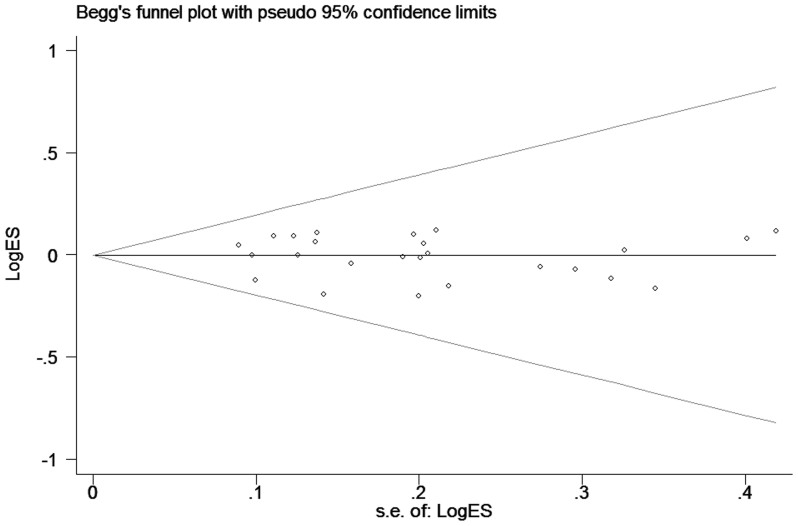
Funnel plot analysis to detect publication bias for C677T polymorphism by dominant model. Each point represents a separate study for the indicated association.

**Figure 4:**
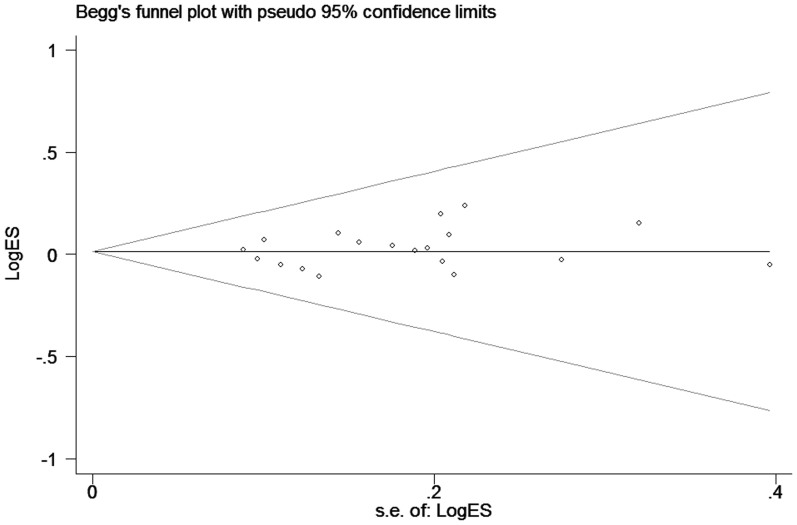
Funnel plot analysis to detect publication bias for A1298C polymorphism by dominant model. Each point represents a separate study for the indicated association.

The shape of the funnel plots seemed asymmetry for the C677T and A1298C polymorphisms (Figure 3 and Figure 4), and we did not detect any significant publication bias by the Egger's test for C677T polymorphism (homozygous: *P* = 0.802; heterozygous: *P* = 0.462; recessive model: *P* = 0.667; dominant model: *P* = 0.568 and allele comparing: *P* = 0.761), and A1298C polymorphism (homozygous: *P* = 0.195; heterozygous: *P* = 0.767; recessive model: *P* = 0.274; dominant model: *P* = 0.312 and allele comparing: *P* = 0.152).

## Discussion

To our knowledge, the current meta-analysis is the largest one to investigate the association between *MTHFR* gene polymorphisms and NHL risk. Pooled analysis for the C677T polymorphism contained 25 studies with a total of 7448 NHL patients and 11146 controls; meanwhile, pooled analysis for the A1298C polymorphism encompassed 19 studies with 6173 NHL patients and 9725 controls. The meta-analysis observed no significant association between *MTHFR* C677T and A1298C polymorphisms and overall NHL risk. However, stratified analyses by ethnicity revealed that the C677T polymorphism increased NHL risk for Caucasians but decreased risk for Asians. The study of the same SNP also observed a significantly increased risk of FL, but not DLBCL, when the analysis was stratified by subtype of NHL. Moreover, the A1298C polymorphism was associated with increased risk for Asians, while no effect was observed for other ethnic groups. Interestingly, we found the *MTHFR* mRNA expression levels was slightly increased in the Asians carrying 677T alleles (*P* = 0.052), which was in accordance with our findings that C677T polymorphism was significantly associated with decreased NHL risk in the Asian group. Moreover, we also found the 1298C carriers showed significantly decreased *MTHFR* mRNA expression (*P* = 0.028), which corresponded to the evidence of association of 1298C polymorphism with increased NHL risk. Therefore, this results suggested that our findings from association studies for Asians may be biological plausible. *MTHFR* gene variants play an important role in the outcome of NHL patients. Examination of polymorphisms in the folate pathway genes might facilitate to reduce chemotherapy toxicity and improve survival by indicating when dose adjustments or alternative treatments are needed^40^.

Folate is a critical nutrient and coenzyme involved in DNA synthesis and methylation, and folate deficiency has been reported associated with numerous malignancies^43^. The product of *MTHFR* gene plays an important role in the methylation of homocysteine into methionine, sequentially leading to DNA methylation^44^. *MTHFR* C677T and A1298C polymorphisms were widely investigated in varieties of cancers. The former genetic variation is located in exon 4, and can lead to amino acid change from alanine to valine, which was first reported in 1995. This variation was reported to associate with reduced enzyme activity from a total of 40 subjects, with the CT and TT genotypes having ~60% and ~30% of the wild-type enzyme activity, respectively^45^. The A1298C polymorphism is located in exon 7. It has also been reported the CC genotype carriers having ~60% of wild-type enzyme activity^46^. Some of the previous studies failed to found this association^21^,^47^. Based on the largest meta-analysis to date, none of these two polymorphisms of interest was associated with overall NHL risk. Nonetheless, stratification analyses by ethnicity detected that analyzed SNPs significantly altered the risk of developing NHL in different ethnic groups. SNPexp online tool allows us to evaluate genotypes of *MTHFR* C677T and A1298C polymorphisms and their respective *MTHFR* transcript expression levels. With this in mind, we further investigated whether the biological results are in accordance with the observed association. We performed genotype-based mRNA expression analysis using the data from 270 individual with three ethnicities. We did not find a similar trend in the mRNA expression for the Caucasians but for the Asians, which may be due to the fact that the genotype counts for the homozygous variants is relatively small.

As so far, only two meta-analyses, which were nested in case-control studies, have investigated the association of *MTHFR* C677T and/or A1298C polymorphisms and NHL susceptibility. The study carried out by Lee et al.^28^ only studied the C677T polymorphism in Caucasians, consisting 13 studies with a total of 4245 cases and 5594 controls. The study observed increased NHL risk for T variant allele carrier in the Caucasians. Subjects with T alleles showed similarly increased risk of DLBCL and FL, in the subgroup analysis by NHL subtype. Another study included 4176 cases and 7585 controls for C677T, as well as 3648 cases and 6331 controls for A1298C polymorphism, in which no significant associations were observed for all subjects^37^. Some of the significant findings described above were not validated in our meta-analysis. For instance, the finding that the *MTHFR* C677T polymorphism was associated with elevated risk for DLBCL in homozygous model^28^ could not be duplicated in our study. Such associations were no longer significant in the current meta-analysis upon the inclusion of seven more studies. We also found some significant associations that were not observed in the previous studies, one example of which was that we found the C677T polymorphism decreased NHL risk for Asians, whereas the A1298C polymorphism conferred an increased risk to them. These new findings may be ascribed to the inclusion of more investigations with much large sample size in the current meta-analysis. It was noteworthy that we found the C677T polymorphism was associated with increased NHL risk for Caucasians while with decreased NHL susceptibility for Asians. The opposite findings in different ethnic groups may be resulted from ethnicity difference as well as the number of investigations. Earlier studies indicated that diet of Western contains high heterocyclic amines and polycyclic aromatic hydrocarbons^48^,^49^, which is relatively low in the diets of other ethnicities, which may contribute to the different effects of C677T polymorphism on cancer risk. Though we have included the latest investigations as well as publications written in Chinese, the current meta-analysis still has several limitations to be addressed. First, the sample size of cases from most eligible studies is relatively limited (<500), except for five studies^21^,^23^,^28^,^29^,^32^. Among the five studies, only one study has case more than one thousand^29^. The relative limited cases may have compromised statistical power. Second, we only included the studies written in English and Chinese, and might miss some investigations written in other languages. Third, heterogeneity was observed under some genetic models, so the results should be interpreted cautiously. Finally, our results were derived from unadjusted estimates due to lack of the original data, such as age, gender, smoking, drinking and dietary intaking habits, occupational exposures, as well as virus infections.

In summary, this study indicated that C677T and A1298C polymorphisms in the *MTHFR* gene may be associated with NHL susceptibility, especially for Caucasians, Asians and FL. Well-designed prospective studies with large sample size should be conducted to validate our findings.

## Methods

### Literature search strategy

We identified publications examined the association between *MTHFR* gene polymorphisms and NHL from MEDLINE and EMBASE using the following search items: “*MTHFR* or *methylenetetrahydrofolate reductase*”, “polymorphism or variant or variation” and “non-Hodgkin lymphoma or non-Hodgkin's lymphoma or NHL” (the last search updated was on January 8, 2014). We also identified related publications written in Chinese from Chinese Biomedical (CBM) database using the combinations terms of “*MTHFR*”, “NHL” and “polymorphism” in Chinese. Besides, we identified additional studies by searching of the references from retrieved studies manually. We only included the latest or the largest sample size studies in our final meta-analysis, if there exists more than one article published using the same subjects or overlapping data.

### Inclusion and exclusion criteria

Studies included in the final meta-analysis had to meet the following criteria: (1) evaluate *MTHFR* C677T and/or A1298C polymorphisms and NHL risk; (2) be a case-control study, nested case-control study or a cohort study; (3) written in English or Chinese; (4) contain SNP genotype data; (5) independent from other studies; (6) provide sufficient data to calculate odds ratios (ORs) and their corresponding 95% confidence intervals (CIs).

The studies were excluded if genotype frequency data in the controls for *MTHFR* C677T and A1298C polymorphisms demonstrated departure from Hardy-Weinberg equilibrium (HWE) without further evidence showing that genotype distribution of other SNPs in controls followed HWE. In addition, case-only studies, case reports, conference abstract, reviews, meta-analyses and studies without detailed data were excluded.

### Data extraction

Two investigators (JH and XL) independently extracted the following information from all eligible studies according to the inclusion and exclusion criteria: the first author's surname, year of publication, country of origin, ethnicity, cancer type and subtype (FL and DLBCL), control source (population based or hospital based), the total number of cases and controls, genotyping methods, minor allele frequency (MAF) for controls, *P* values for HWE for the control subjects and numbers of cases and controls with the CC, CT and TT genotypes for the C677T polymorphism and AA, AC and CC genotypes for the A1298C polymorphism. Any disagreement was resolved by discussion within our team numbers until consensus was reached.

### Genotype based mRNA expression analysis

The genotypes data for *MTHFR* C677T and A1298C polymorphisms were available from HapMap (http://hapmap.ncbi.nlm.nih.gov/) for 270 subjects with three different ethnicities and their corresponding mRNA expression levels data were available from SNPexp (http://app3.titan.uio.no/biotools/tool.php?app=snpexp) as described previously^50^,^51^,^52^,^53^.

### Quality assessment

Two investigators assessed the quality of each investigation using the quality assessment criteria (Supplemental Table 2), which was derived from previously published meta-analysis of molecular association studies^54^. Quality scores of studies ranged from 0 (lowest) to 15 (highest). Studies with scores ≤ 9 were categorized into low quality, while those with scores > 9 were considered as high quality. A third investigator (JZ) would be involved if there existed any disagreement.

### Statistical methods

Crude ORs and their corresponding 95% CIs were used to evaluate the strength of associations between *MTHFR* gene polymorphisms and NHL risk. The pooled ORs were estimated for C677T polymorphism under the homozygous model (TT vs. CC), heterozygous model (CT vs. CC), recessive model (TT vs. CT + CC), dominant model (CT + TT vs. CC) and allele comparison (T vs. C). The same genetic models were also adopted for A1298C polymorphism as followed: homozygous model (CC vs. AA), heterozygous model (AC vs. AA), recessive model (CC vs. AC + AA), dominant model (AC + CC vs. AA) and allele comparison (C vs. A). Goodness-of-fit chi-square test was used to test deviation from HWE for the genotypes of controls. *P* < 0.05 was considered significant. The Chi-square based Q-test was performed to evaluate the heterogeneity across the studies. The random-effects model^55^ was chosen when significant heterogeneous exist (*P_heterogeneity_* < 0.10); otherwise, fixed-effects model (the Mantel–Haenszel method)^56^ would be adopted. Stratification and meta-regression analyses were conducted to explore the potential source of heterogeneity across studies. Furthermore, stratification analyses were conducted by ethnicity (i.e., Asians, Caucasians, and Mixed that contained more than one ethnic group), control source (hospital-based and population-based), quality score of studies (low and high), and tumor subtype (FL and DLBCL). Sensitivity analysis was performed to assess the stability of the results by sequentially excluding one study at a time and recalculating the pooled ORs and their corresponding 95% CIs. Furthermore, both the Begg's funnel plot^57^ and the Egger's linear regression test^58^ were performed to assess the potential publication bias. The differences in mRNA expression levels for different genotypes were evaluated by one-way ANOVA, and the mRNA expression levels trend were evaluated by General linear model. All statistical tests were performed with STATA software (version 11.0; Stata Corporation, College Station, TX) and SAS software (version 9.1; SAS Institute, Cary, NC). All the statistics were two-sided, and *P* < 0.05 was considered as significant findings.

## Supplementary Material

Supplementary Information Dataset 1

## Figures and Tables

**Table 1:** Characteristics of studies included in the current meta-analysis

Surname	Year	Country	Ethnicity	Source	Genotype method	Case				Control				MAF	HWE	Score
						11	12	22	All	11	12	22	All			
**C677T polymorphism**																
Gonzalez Ordonez	2000	Spain	Caucasian	HB	PCR-RFLP	21	21	5	47	92	88	20	200	0.32	0.876	6
Lincz	2003	Australia	Caucasian	HB	PCR-RFLP	73	58	17	148	145	133	21	299	0.29	0.198	7
Toffoli	2003	Italy	Caucasian	PB	PCR-RFLP	44	49	18	111	147	233	85	465	0.43	0.662	11
Gemmati	2004	Italy	Caucasian	PB	PCR-RFLP	60	101	39	200	78	128	51	257	0.45	0.908	10
Linnebank	2004	German	Caucasian	PB	PCR-RFLP	13	12	6	31	66	52	24	142	0.35	0.019	8
Matsuo	2004	Japan	Asian	HB	PCR-RFLP	165	122	63	350	182	230	88	500	0.41	0.301	8
Rudd	2004	UK	Caucasian	HB	Taqman	361	381	90	832	383	397	106	886	0.34	0.841	12
Skibola	2004	USA	Caucasian	PB	Taqman	122	160	52	334	288	350	84	722	0.36	0.149	14
Lightfoot	2005	UK	Caucasian	PB	Taqman	247	270	72	589	356	316	83	755	0.32	0.309	14
Stanulla	2005	German	Caucasian	PB	PCR-RFLP	207	216	64	487	184	152	43	379	0.31	0.179	9
Chen	2006	China	Asian	HB	Taqman	11	13	4	28	72	66	19	157	0.33	0.522	8
Deligezer	2006	Turkey	Caucasian	HB	Taqman	31	30	5	66	66	72	16	154	0.34	0.574	9
Niclot	2006	France	Caucasian	PB	DHPLC	66	86	20	172	92	88	24	204	0.33	0.674	8
Timuragaoglu	2006	Turkey	Caucasian	PB	Realtime PCR	31	22	5	58	36	36	10	82	0.34	0.829	9
Lee	2007	Australia	Caucasian	PB	Taqman	253	227	74	554	256	190	57	503	0.30	0.019	11
Lim	2007	USA	Mixed	PB	Taqman	499	477	127	1103	443	396	86	925	0.31	0.853	15
Siraj	2007	Saudi Arabia	Caucasian	PB	PCR-RFLP	109	45	6	160	372	126	13	511	0.15	0.553	10
Gra	2008	Russia	Caucasian	HB	Hybridization	39	28	9	76	85	79	13	177	0.30	0.354	8
Kim	2008	Korea	Asian	PB	PCR-RFLP	223	286	75	584	540	863	297	1700	0.43	0.133	12
Berglund	2009	Sweden	Caucasian	PB	Illumina	154	85	24	263	241	157	32	430	0.26	0.363	10
Ismail	2009	Jordan	Caucasian	PB	PCR-RFLP	34	19	2	55	94	66	10	170	0.25	0.722	10
Wang	2009	Jamaica	Mixed	PB	Taqman	329	58	5	392	204	57	5	266	0.13	0.664	14
Kurzwelly	2010	German	Caucasian	PB	PCR-RFLP	78	81	26	185	96	96	20	212	0.32	0.568	10
Weiner	2011	Russia	Caucasian	PB	Taqman	72	60	11	143	242	198	46	486	0.30	0.553	8
Li	2013	USA	Mixed	PB	Taqman	202	206	72	480	236	246	82	564	0.36	0.173	15
**A1298C polymorphism**																
Lincz	2003	Australia	Caucasian	HB	PCR-RFLP	64	68	13	145	124	139	31	294	0.34	0.385	7
Toffoli	2003	Italy	Caucasian	PB	PCR-RFLP	54	44	13	111	200	222	43	465	0.33	0.094	11
Gemmati	2004	Italy	Caucasian	PB	PCR-RFLP	96	90	14	200	126	110	21	257	0.30	0.659	10
Linnebank	2004	German	Caucasian	PB	PCR-RFLP	16	12	3	31	69	54	19	142	0.32	0.116	9
Matsuo	2004	Japan	Asian	HB	PCR-RFLP	209	122	19	350	327	150	23	500	0.20	0.282	8
Rudd	2004	UK	Caucasian	HB	Taqman	397	363	72	832	412	389	85	886	0.32	0.622	12
Skibola	2004	USA	Caucasian	PB	Taqman	178	128	27	333	341	310	71	722	0.31	0.964	14
Lightfoot	2005	UK	Caucasian	PB	Taqman	288	250	51	589	347	331	77	755	0.32	0.882	14
Niclot	2006	France	Caucasian	PB	DHPLC	79	76	17	172	102	81	15	198	0.28	0.844	8
Lim	2007	USA	Mixed	PB	Taqman	540	480	104	1124	461	393	81	935	0.30	0.831	15
Siraj	2007	Saudi Arabia	Caucasian	PB	PCR-RFLP	38	40	35	113	239	220	52	511	0.32	0.896	10
Gra	2008	Russia	Caucasian	HB	Hybridization	36	30	10	76	81	82	14	177	0.31	0.278	8
Kim	2008	Korea	Asian	PB	Taqman	372	182	29	583	1147	500	53	1700	0.18	0.868	12
Berglund	2009	Sweden	Caucasian	PB	Illumina	116	121	25	262	214	196	39	449	0.31	0.533	10
Ismail	2009	Jordan	Caucasian	PB	PCR-RFLP	20	23	12	55	76	81	13	170	0.31	0.172	10
Wang	2009	Jamaica	Mixed	PB	Taqman	277	98	15	390	201	65	9	275	0.15	0.198	14
Kurzwelly	2010	German	Caucasian	PB	PCR-RFLP	72	96	17	185	106	89	17	212	0.29	0.779	10
Weiner	2011	Russia	Caucasian	PB	Taqman	59	52	22	133	232	215	56	503	0.33	0.562	8
Li	2013	USA	Mixed	PB	Taqman	246	203	40	489	265	250	59	574	0.32	0.997	15

HB, Hospital based; PB, Population based; PCR-RFLP, Polymorphism chain reaction-restriction fragment length polymorphism; DHPLC, Denaturing high performance liquid chromatography; MAF, Minor allele frequency; HWE, Hardy-Weinberg equilibrium.

**Table 2:** Meta-analysis of the association between *MTHFR* C677T and A1298C polymorphisms and cancer risk

Variables	No. of study	Sample	Homozygous		Heterozygous		Recessive		Dominant		Allele Comparing	
		Size	OR (95% CI)	*P* ^het^	OR (95% CI)	*P* ^het^	OR (95% CI)	*P* ^het^	OR (95% CI)	*P* ^het^	OR (95% CI)	*P* ^het^
C677T (rs1801133)			TT vs. CC		CT vs. CC		TT vs. (CT + CC)		(CT + TT) vs. CC		T vs. C	
All	25	7448/11146	1.06 (0.93–1.20)	0.092	0.97 (0.89–1.07)	0.027	1.04 (0.95–1.15)	0.465	0.99 (0.90–1.08)	0.006	1.01 (0.94–1.08)	0.006
Ethnicity												
Caucasian	19	4511/7034	**1.15 (1.01–1.30)**	0.720	1.06 (0.97–1.15)	0.639	1.11 (0.98–1.25)	0.838	1.08 (0.99–1.16)	0.546	**1.07 (1.01–1.13)**	0.556
Asian	3	962/2357	**0.70 (0.54–0.91)**	0.320	0.74 (0.55–1.01)	0.108	0.81 (0.66–1.00)	0.187	**0.74 (0.59–0.91)**	0.238	**0.81 (0.72–0.90)**	0.391
Mixed	3	1975/1755	1.16 (0.92–1.46)	0.364	0.92 (0.71–1.19)	0.067	1.15 (0.93–1.43)	0.473	0.93 (0.71–1.22)	0.034	0.96 (0.76–1.20)	0.023
Source of control												
PB	18	5901/8773	1.08 (0.92–1.27)	0.050	1.01 (0.92–1.12)	0.086	1.05 (0.94–1.17)	0.329	1.02 (0.92–1.13)	0.010	1.02 (0.94–1.11)	0.003
HB	7	1547/2373	0.95 (0.77–1.17)	0.552	0.86 (0.69–1.06)	0.120	1.02 (0.84–1.25)	0.560	0.89 (0.75–1.05)	0.244	0.95 (0.86–1.04)	0.571
Score												
Low	11	1606/2780	1.04 (0.85–1.28)	0.650	0.95 (0.78–1.16)	0.048	1.09 (0.89–1.32)	0.876	0.97 (0.81–1.17)	0.078	1.00 (0.90–1.12)	0.319
High	14	5842/8366	1.06 (0.89–1.27)	0.018	0.99 (0.90–1.09)	0.092	1.03 (0.93–1.15)	0.139	1.00 (0.89–1.11)	0.010	1.01 (0.92–1.11)	0.001
Subtype												
DLBCL	12	1966/7271	1.03 (0.81–1.30)	0.047	0.94 (0.78–1.13)	0.002	1.02 (0.88–1.20)	0.196	0.96 (0.80–1.14)	0.002	1.00 (0.89–1.13)	0.011
FL	9	1251/4508	**1.25 (1.01–1.53)**	0.501	0.93 (0.75–1.14)	0.034	**1.26 (1.04–1.53)**	0.645	0.98 (0.81–1.19)	0.049	1.05 (0.93–1.19)	0.164
A1298C (rs1801131)			CC vs. AA		AC vs. AA		CC vs. (AC + AA)		(AC + CC) vs. AA		C vs. A	
All	19	6173/9725	1.21 (0.97–1.50)	<0.001	1.02 (0.95–1.09)	0.471	1.21 (0.98–1.49)	<0.001	1.05 (0.96–1.14)	0.064	1.08 (0.98–1.18)	<0.001
Ethnicity												
Caucasian	14	3237/5741	1.24 (0.93–1.67)	<0.001	0.98 (0.89–1.08)	0.490	1.25 (0.94–1.66)	<0.001	1.03 (0.92–1.17)	0.075	1.09 (0.96–1.23)	<0.001
Asian	2	933/2200	**1.54 (1.05–2.24)**	0.506	1.17 (0.99–1.39)	0.494	1.46 (1.00–2.11)	0.430	**1.21 (1.03–1.42)**	0.646	**1.20 (1.05–1.38)**	0.908
Mixed	3	2003/1784	0.96 (0.72–1.29)	0.293	1.00 (0.87–1.14)	0.471	0.98 (0.77–1.24)	0.426	0.99 (0.85–1.15)	0.291	0.99 (0.86–1.14)	0.199
Source of control												
PB	15	4770/7868	1.26 (0.97–1.64)	<0.001	1.01 (0.93–1.10)	0.400	1.25 (0.97–1.61)	<0.001	1.06 (0.95–1.18)	0.039	1.09 (0.98–1.22)	<0.001
HB	4	1403/1857	0.98 (0.75–1.28)	0.467	1.02 (0.88–1.19)	0.381	0.99 (0.76–1.27)	0.443	1.02 (0.88–1.19)	0.356	1.02 (0.90–1.15)	0.319
Score												
Low	6	907/1814	1.27 (0.94–1.71)	0.669	1.08 (0.91–1.29)	0.680	1.26 (0.94–1.68)	0.612	1.11 (0.94–1.31)	0.732	1.12 (0.98–1.27)	0.673
High	13	5266/7911	1.21 (0.92–1.59)	<0.001	1.00 (0.93–1.08)	0.299	1.21 (0.93–1.57)	<0.001	1.04 (0.93–1.16)	0.019	1.08 (0.96–1.21)	<0.001
Subtype												
DLBCL	9	1624/6331	1.23 (0.84–1.79)	0.002	1.02 (0.91–1.15)	0.515	1.25 (0.86–1.81)	0.001	1.06 (0.94–1.18)	0.127	1.09 (0.92–1.28)	0.001
FL	8	1039/4023	1.26 (0.88–1.79)	0.081	1.04 (0.90–1.21)	0.403	1.23 (0.90–1.67)	0.153	1.07 (0.93–1.23)	0.147	1.10 (0.93–1.30)	0.036

HB, Hospital based; PB, Population based; DLBCL, diffuse large B-cell lymphoma; FL, follicular lymphoma.

**Table 3:** *MTHFR* mRNA expression by the genotypes of SNPs, using data from the HapMap^a^

Population	C667T (rs1801133)					A1298C (rs1801131)				
	genotypes	No.	Mean ± SD	*P*^b^	*P*_trend_^c^	genotypes	No.	Mean ± SD	*P*^b^	*P*_trend_^c^
CEU	CC	48	6.14 ± 0.10		0.904	AA	41	6.12 ± 0.09		0.139
	CT	37	6.14 ± 0.09	0.924		AC	36	6.15 ± 0.10	0.069	
	TT	5	6.12 ± 0.09	0.685		CC	13	6.16 ± 0.09	0.163	
	Dominant	42	6.14 ± 0.09	0.983		Dominant	49	6.15 ± 0.09	**0.046**	
YRI^d^	CC	70	6.20 ± 0.08		0.329	AA	71	6.20 ± 0.07		0.197
	CT	19	6.19 ± 0.06	0.651		AC	17	6.19 ± 0.09	0.504	
	TT	1	6.09	0.171		CC	1	6.07	0.074	
	Dominant	20	6.19 ± 0.06	0.470		Dominant	18	6.18 ± 0.09	0.317	
Asian^d^	CC	30	6.18 ± 0.08		0.116	AA	59	6.22 ± 0.10		0.074
	CT	40	6.21 ± 0.09	0.120		AC	28	6.17 ± 0.09	**0.024**	
	TT	19	6.23 ± 0.11	0.052		CC	3	6.20 ± 0.06	0.814	
	Dominant	59	6.22 ± 0.10	0.057		Dominant	31	6.17 ± 0.08	**0.028**	
All^d^	CC	148	6.17 ± 0.09		0.360	AA	171	6.19 ± 0.09		0.186
	CT	96	6.18 ± 0.09	0.732		AC	81	6.17 ± 0.09	0.107	
	TT	25	6.20 ± 0.12	0.156		CC	17	6.16 ± 0.09	0.261	
	Dominant	121	6.18 ± 0.10	0.422		Dominant	98	6.16 ± 0.09	0.069	

^a^Genotyping data and mRNA expression levels for *MTHFR* by genotypes were obtained from the HapMap phase II release 23 data from EBV-transformed lymphoblastoid cell lines from 270 individuals.

^b^Two-side Student's *t* test within the stratum.

^c^*P* values for the trend test of *MTHFR* mRNA expression among 3 genotypes for each SNP from a general linear model.

^d^There were missing data because genotyping data were not available.
