# Supplementary material for: Association of MTHFR C677T and A1298C polymorphisms with non-Hodgkin lymphoma susceptibility: Evidence from a meta-analysis
Source: Sci Rep. 2014 Aug 22;4:6159. doi: 10.1038/srep06159 (PMC5381410; doi:10.1038/srep06159)
Supplement: Supplementary Information — Dataset 1 [file srep06159-s1.doc]

**Association of *MTHFR* C677T and A1298C polymorphisms with non-Hodgkin lymphoma susceptibility: Evidence from a meta-analysis**

Jing He ^1^, Xiao-Yu Liao ^1^, Jin-Hong Zhu ^2^, Wen-Qiong Xue ^1^, Guo-Ping Shen ^3^, Shao-Yi Huang ^1^, Wei Chen ^4^, Wei-Hua Jia ^1,*^

1 State Key Laboratory of Oncology in South China, Department of Experimental Research, Collaborative Innovation Center for Cancer Medicine, Sun Yat-Sen University Cancer Center, Guangzhou, Guangdong 510060, China

2 Molecular Epidemiology Laboratory and Laboratory Medicine, Harbin Medical University Cancer Hospital, Harbin, Heilongjiang 150040, China

3 Department of Radiation Oncology, The First Affiliated Hospital, Sun Yat-Sen University, Guangzhou, Guangdong 510080, China

4 Department of Neurosurgery, The Second Affiliated Hospital, Sun Yat-Sen University, Guangzhou, Guangdong 510120, China

***Correspondence to：**Wei-Hua Jia, State Key Laboratory of Oncology in South China, Department of Experimental Research, Collaborative Innovation Center for Cancer Medicine, Sun Yat-Sen University Cancer Center, 651 Dongfengdong Road，Guangzhou, Guangdong 510060, China, Tel.: (+86-20) 87342327; Fax: (+86-20) 87343392, E-mail: jiaweih@mail.sysu.edu.cn.

| **Supplemental Table 1.** The frequency distribution of *MTHFR* polymorphisms for DLBCL and FL included in the current meta-analysis | | | | | | | | | | | | | | |
| --- | --- | --- | --- | --- | --- | --- | --- | --- | --- | --- | --- | --- | --- | --- |
| Surname | Year | Subtype | Country | Ethnicity | Source | Genotype method | Case | | | | Control | | | |
|  |  |  |  |  |  |  | 11 | 12 | 22 | All | 11 | 12 | 22 | All |
| **C677T polymorphism** | | | | | | | | | | | | | | |
| Lincz | 2003 | DLBCL | Australia | Caucasian | HB | PCR-RFLP | 37 | 25 | 10 | 72 | 145 | 133 | 21 | 299 |
| Lincz | 2003 | FL | Australia | Caucasian | HB | PCR-RFLP | 21 | 20 | 6 | 47 | 145 | 133 | 21 | 299 |
| Toffoli | 2003 | DLBCL | Italy | Caucasian | PB | PCR-RFLP | 44 | 49 | 18 | 111 | 147 | 233 | 85 | 465 |
| Matsuo | 2004 | DLBCL | Japan | Asian | HB | PCR-RFLP | 61 | 37 | 25 | 123 | 182 | 230 | 88 | 500 |
| Matsuo | 2004 | FL | Japan | Asian | HB | PCR-RFLP | 38 | 23 | 13 | 74 | 182 | 230 | 88 | 500 |
| Skibola | 2004 | DLBCL | USA | Caucasian | PB | Taqman | 37 | 51 | 19 | 107 | 288 | 350 | 84 | 722 |
| Skibola | 2004 | FL | USA | Caucasian | PB | Taqman | 44 | 55 | 23 | 122 | 288 | 350 | 84 | 722 |
| Lightfoot | 2005 | DLBCL | UK | Caucasian | PB | Taqman | 119 | 118 | 33 | 270 | 356 | 316 | 83 | 755 |
| Lightfoot | 2005 | FL | UK | Caucasian | PB | Taqman | 82 | 101 | 24 | 207 | 356 | 316 | 83 | 755 |
| Stanulla | 2005 | DLBCL | German | Caucasian | PB | PCR-RFLP | 31 | 28 | 6 | 65 | 184 | 152 | 43 | 379 |
| Niclot | 2006 | FL | France | Caucasian | PB | DHPLC | 66 | 86 | 20 | 172 | 92 | 88 | 24 | 204 |
| Timuragaoglu | 2006 | DLBCL | Turkey | Caucasian | PB | Realtime PCR | 31 | 22 | 5 | 58 | 36 | 36 | 10 | 82 |
| Lee | 2007 | DLBCL | Australia | Caucasian | PB | Taqman | 77 | 85 | 17 | 179 | 256 | 190 | 57 | 503 |
| Lee | 2007 | FL | Australia | Caucasian | PB | Taqman | 107 | 69 | 33 | 209 | 256 | 190 | 57 | 503 |
| Lim | 2007 | DLBCL | USA | Mixed | PB | Taqman | 152 | 156 | 35 | 343 | 443 | 396 | 86 | 925 |
| Lim | 2007 | FL | USA | Mixed | PB | Taqman | 131 | 100 | 33 | 264 | 443 | 396 | 86 | 925 |
| Siraj | 2007 | DLBCL | Saudi Arabia | Caucasian | PB | PCR-RFLP | 109 | 45 | 6 | 160 | 372 | 126 | 13 | 511 |
| Kim | 2008 | DLBCL | Korea | Asian | PB | PCR-RFLP | 131 | 144 | 41 | 316 | 540 | 863 | 297 | 1700 |
| Berglund | 2009 | DLBCL | Sweden | Caucasian | PB | Illumina | 89 | 56 | 17 | 162 | 241 | 157 | 32 | 430 |
| Berglund | 2009 | FL | Sweden | Caucasian | PB | Illumina | 65 | 29 | 7 | 101 | 241 | 157 | 32 | 430 |
| Ismail | 2009 | FL | Jordan | Caucasian | PB | PCR-RFLP | 34 | 19 | 2 | 55 | 94 | 66 | 10 | 170 |
| **A1298C polymorphism** | | | | | | | | | | | | | | |
| Lincz | 2003 | DLBCL | Australia | Caucasian | HB | PCR-RFLP | 31 | 31 | 8 | 70 | 124 | 139 | 31 | 294 |
| Lincz | 2003 | FL | Australia | Caucasian | HB | PCR-RFLP | 22 | 19 | 3 | 44 | 124 | 139 | 31 | 294 |
| Toffoli | 2003 | DLBCL | Italy | Caucasian | PB | PCR-RFLP | 54 | 44 | 13 | 111 | 200 | 222 | 43 | 465 |
| Matsuo | 2004 | DLBCL | Japan | Asian | HB | PCR-RFLP | 75 | 41 | 7 | 123 | 327 | 150 | 23 | 500 |
| Matsuo | 2004 | FL | Japan | Asian | HB | PCR-RFLP | 38 | 29 | 7 | 74 | 327 | 150 | 23 | 500 |
| Skibola | 2004 | DLBCL | USA | Caucasian | PB | Taqman | 59 | 38 | 9 | 106 | 341 | 310 | 71 | 722 |
| Skibola | 2004 | FL | USA | Caucasian | PB | Taqman | 63 | 49 | 10 | 122 | 341 | 310 | 71 | 722 |
| Lightfoot | 2005 | DLBCL | UK | Caucasian | PB | Taqman | 128 | 120 | 22 | 270 | 347 | 331 | 77 | 755 |
| Lightfoot | 2005 | FL | UK | Caucasian | PB | Taqman | 103 | 84 | 20 | 207 | 347 | 331 | 77 | 755 |
| Niclot | 2006 | FL | France | Caucasian | PB | DHPLC | 79 | 76 | 17 | 172 | 102 | 81 | 15 | 198 |
| Lim | 2007 | DLBCL | USA | Mixed | PB | Taqman | 168 | 152 | 32 | 352 | 461 | 393 | 81 | 935 |
| Lim | 2007 | FL | USA | Mixed | PB | Taqman | 125 | 115 | 27 | 267 | 461 | 393 | 81 | 935 |
| Siraj | 2007 | DLBCL | Saudi Arabia | Caucasian | PB | PCR-RFLP | 38 | 40 | 35 | 113 | 239 | 220 | 52 | 511 |
| Kim | 2008 | DLBCL | Korea | Asian | PB | Taqman | 201 | 103 | 11 | 315 | 1147 | 500 | 53 | 1700 |
| Berglund | 2009 | DLBCL | Sweden | Caucasian | PB | Illumina | 74 | 74 | 16 | 164 | 214 | 196 | 39 | 449 |
| Berglund | 2009 | FL | Sweden | Caucasian | PB | Illumina | 42 | 47 | 9 | 98 | 214 | 196 | 39 | 449 |
| Ismail | 2009 | FL | Jordan | Caucasian | PB | PCR-RFLP | 20 | 23 | 12 | 55 | 76 | 81 | 13 | 170 |
| DLBCL, Diffuse large B-cell lymphoma; FL, Follicular lymphoma; HB, Hospital based; PB, Population based; PCR-RFLP, Polymorphism chain reaction-restriction fragment length polymorphism; DHPLC, Denaturing high performance liquid chromatography. | | | | | | | | | | | | | | |

| Supplemental Table 2. Score of quality assessment | |
| --- | --- |
| **Criteria** | **Score** |
| Representativeness of case |  |
| Selected from population cancer registry | 2 |
| Selected from hospital | 1 |
| No method of selection described | 0 |
| Representativeness of control |  |
| Population-based | 3 |
| Blood donors | 2 |
| Hospital-based | 1 |
| Not described | 0 |
| Ascertainment of NHL case |  |
| Histopathologic confirmation | 2 |
| by patient medical record | 1 |
| Not described | 0 |
| Control selection |  |
| Controls matched with cases by age and sex | 2 |
| Controls matched with cases only by age or by sex | 1 |
| Not matched or not descried | 0 |
| Genotyping examination |  |
| Genotyping done blindly and quality control | 2 |
| Only genotyping done blindly or quality control | 1 |
| Unblinded and without quality control | 0 |
| HWE |  |
| HWE in the control group | 1 |
| HWD in the control group or not mentioned | 0 |
| Total sample size |  |
| > 1000 | 3 |
| 501 - 1000 | 2 |
| 201 - 500 | 1 |
| ≤ 200 | 0 |
